# Clinical and inflammatory characteristics of Asthma-COPD overlap in workers with occupational asthma

**DOI:** 10.1371/journal.pone.0193144

**Published:** 2018-03-02

**Authors:** Iñigo Ojanguren, Gregory Moullec, Jad Hobeika, Marc Miravitlles, Catherine Lemiere

**Affiliations:** 1 Centre intégré universitaire de santé et de services sociaux du Nord-de-l'Île-de-Montréal, Hôpital du Sacré-Cœur de Montréal, Université de Montréal, Montréal, Quebec, Canada; 2 CIBER Enfermedades Respiratorias (CIBERES), Instituto de Salud Carlos III, Barcelona, Spain; 3 Public Health School, Department of Social and Preventive Medicine, University of Montreal, Quebec, Canada; 4 Centre intégré de santé et de services sociaux des Laurentides, Hôpital Saint-Eustache, Saint-Eustache, Québec, Canada; 5 Servicio de Neumología, Hospital Universitari Vall d'Hebron, Barcelona, España; National and Kapodistrian University of Athens, GREECE

## Abstract

**Introduction:**

Although Asthma-COPD Overlap (ACO) has been described among populations of subjects with COPD or asthma, ACO has never been described among a population of subjects with occupational asthma (OA).

**Objectives:**

The aims of this study were to: 1. identify ACO in a population of subjects with OA; and 2. compare the clinical characteristics between ACO and OA.

**Methods:**

This retrospective study included all subjects diagnosed with OA between 2000 and 2017 in an OA referral center. Occupational Asthma-COPD Overlap (OACO) was defined as post-bronchodilator FEV_1_/FVC < 70% and smoking history ≥ 10 pack-years, along with a diagnosis of OA.

**Results:**

Three hundred and four subjects were included, 262 (86.2%) were classified as OA and 42 (13.8%) as OACO. OA subjects presented higher sputum eosinophil counts after a specific-inhalation challenge than subjects with OACO (median [IQR]: 6.5 [17.0] vs 2.3 [3.5]). After adjusting for confounding factors, subjects with OACO were older (OR: 1.10 [1.05; 1.14]) and were taking higher doses of inhaled corticosteroids than OA subjects (OR, 5.20 [1.77; 16.48]). Subjects with OACO were less often atopic than OA subjects (OR, 0.19 [0.07; 0.62]).

**Conclusions:**

Subjects with OACO constitute a distinct clinical and inflammatory phenotype from subjects with OA.

## Introduction

Asthma and COPD have been described as two distinct diseases that often overlap[1]. Several national and international societies have recently defined the Asthma-COPD Overlap (ACO). The Global Initiative for Asthma (GINA) and Global Initiative for Chronic Obstructive Lung Disease (GOLD) have proposed a consensus document aiming to improve the characterization and identification of these patients[2]. ACO subjects are usually described as asthmatics with smoking history who develop non-fully reversible airflow limitation or subjects with COPD who show features of asthma[3].

Clinical, functional and inflammatory characteristics of ACO have been described within large cohorts of COPD subjects, where the diagnosis of asthma was performed a posteriori, based on highly reversible airflow obstruction, Th2-type of inflammation and/or previously reported physician diagnosis of asthma[4–7]. In general, subjects with ACO are younger, show greater airway hyperresponsiveness, and higher blood and sputum eosinophil counts compared to COPD patients. The identification of ACO within COPD populations allowed the identification of subjects with underlying eosinophilic inflammation who may respond better to inhaled corticosteroids (ICS) than COPD subjects without ACO[8,9]. The relevance of the use of biomarkers such as atopy, IgE, or eosinophilia in blood or sputum has been emphasized to identify ACO subjects among populations of COPD subjects[10–12].

In contrast, the description of ACO subjects among asthmatic populations has been scarce[13–16]. According to the few studies describing ACO subjects among the asthmatic cohorts[13], the characteristics of this phenotype seem to differ from other asthmatics, in terms of demographic characteristics and airway inflammatory patterns: ACO subjects tended to be older, were mainly men, had a lower FEV_1_, and show predominantly a neutrophilic airway inflammation compared with subjects with asthma alone.

Allergic Occupational Asthma (OA) is caused by the exposure of a specific agent at the workplace[17]. Subjects with OA usually include a large proportion of smokers. However, to our knowledge, no study has attempted to describe ACO subjects within cohorts of subjects with OA.

The aims of this study were to: 1. identify ACO in a population of subjects with OA; and 2. compare the clinical characteristics between ACO subjects and subjects with OA.

## Methods

### Study design

Retrospective study using a database of 695 subjects referred for possible OA and investigated at the *Hôpital du Sacré-Coeur de Montréal* (Montreal, Qc, Canada) between 2000 and 2017. The study was approved by the Research Ethics Committee of *Hôpital du Sacré-Coeur de Montréal*. Patients provided informed written consent authorizing to access their medical records for collecting the clinical data, and entering them in the central database. All data were subsequently anonymized before performing the statistical analysis. The researcher who analyzed the data did not have access to the nominal data.

### Subjects

All subjects who had a final diagnosis of OA using a specific-inhalation challenge (SIC) between 2000 and 2017 were included. Subjects with incompletely reversible airflow limitation (post-bronchodilator FEV_1_/FVC less than 70%), a smoking history greater than 10 pack-years, along with a diagnosis of OA were defined as Occupational Asthma-COPD Overlap (OACO)[3,18]. The medical charts of all subjects were reviewed and demographic data such as sex, age, smoking habit, atopy, occupational agents, duration of exposure to the agents, and dosage of ICS at diagnosis were recorded. Sputum differential cell counts and a methacholine challenge were performed before and after exposure to the offending agent during the SIC.

### Atopy and smoking status

Patients were considered atopic if they had at least one positive skin-prick test to any common environmental allergen; twelve different environmental allergens were tested. Non-smokers were defined as subjects who had never smoked; ex-smokers were those who had not smoked for at least six months. The number of pack-years was calculated.

### Spirometry and methacholine challenge

Spirometry with bronchodilator test were performed in accordance with the standards from the American Thoracic Society/European Respiratory Society[19]. Airway responsiveness was assessed by doubling concentrations of methacholine using a Wright nebulizer (output 0.14mL/min) at tidal volumes for two-minute periods. The results were expressed as the concentration of methacholine required to induce a 20% decrease in FEV_1_ (PC_20_)[20].

### Sputum induction and processing

Sputum was induced using inhalations of hypertonic saline at increasing concentrations (3%, 4%, and 5) and was processed as previously described[21]. Briefly, all portions of the sample collected that macroscopically looked unlike saliva were selected and were treated with Sputalysin, followed by Dulbecco’s phosphate-buffered saline. The resulting suspension was then filtered, cytospins were prepared and stained by the Wright method, and cell differential counts were performed.

### Specific-inhalation challenge

SICs were performed as previously described[22], either at the laboratory or at the workplace. On the first day, subjects were exposed to a sham substance for 30 minutes to ensure adequate asthma control. In the following days, subjects were exposed to increasing doses of the agent suspected of causing OA. When the SICs conducted in the laboratory were negative or when the exposure could not be replicated, subjects were accompanied to their workplace by a respiratory therapist, where spirometry was conducted hourly for 7 hours on two consecutive days. A sustained 20% fall in FEV_1_ was required for SIC to be considered positive. A methacholine challenge and a sputum induction were performed at the end of the control day and at the end of the last day of exposure. Long-acting bronchodilators were withheld 72 hours prior to SIC.

### Data analysis

Demographic and clinical variables were compared between OA and OACO groups using a chi-square test for categorical variables (or the Fisher exact test when one of the cells size was less than 5), and a Student’s t test or Mann-Whitney *U* test for continuous variables, as appropriate. Subjects were divided into four inflammatory phenotypes according to their sputum cell count: eosinophilic (sputum eosinophils > 1.01%), neutrophilic (sputum neutrophils > 61%), mixed granulocytic (sputum eosinophils > 1.01% and sputum neutrophils > 61%), and pauci granulocytic (neutrophils ≤ 61% and sputum eosinophils ≤ 1.01%)[23].

We performed a multivariate logistic regression model in order to identify the factors significantly and independently associated with OACO. Based on exploratory bivariate analyses, the model consisted of the following variables: sex, age, ICS dose, atopy, duration of exposure before consultation, duration of exposure before symptoms. Confidence interval values were estimated using a bootstrapping procedure with bias corrected (R Library boot). To deal with missing data, we performed a multiple imputation procedure and twenty datasets were created (R Library Mice). However, due to the high number of missing data for blood and sputum cell counts as well as PC_20_ in the OACO group, these variables were not taken into account in our prediction model.

All analyses were conducted using R (Version 3.1.1, The R Foundation for Statistical Computing). Two-tailed p-valued at 5% were considered statistically significant.

## Results and discussion

### Results

**Fig 1:**
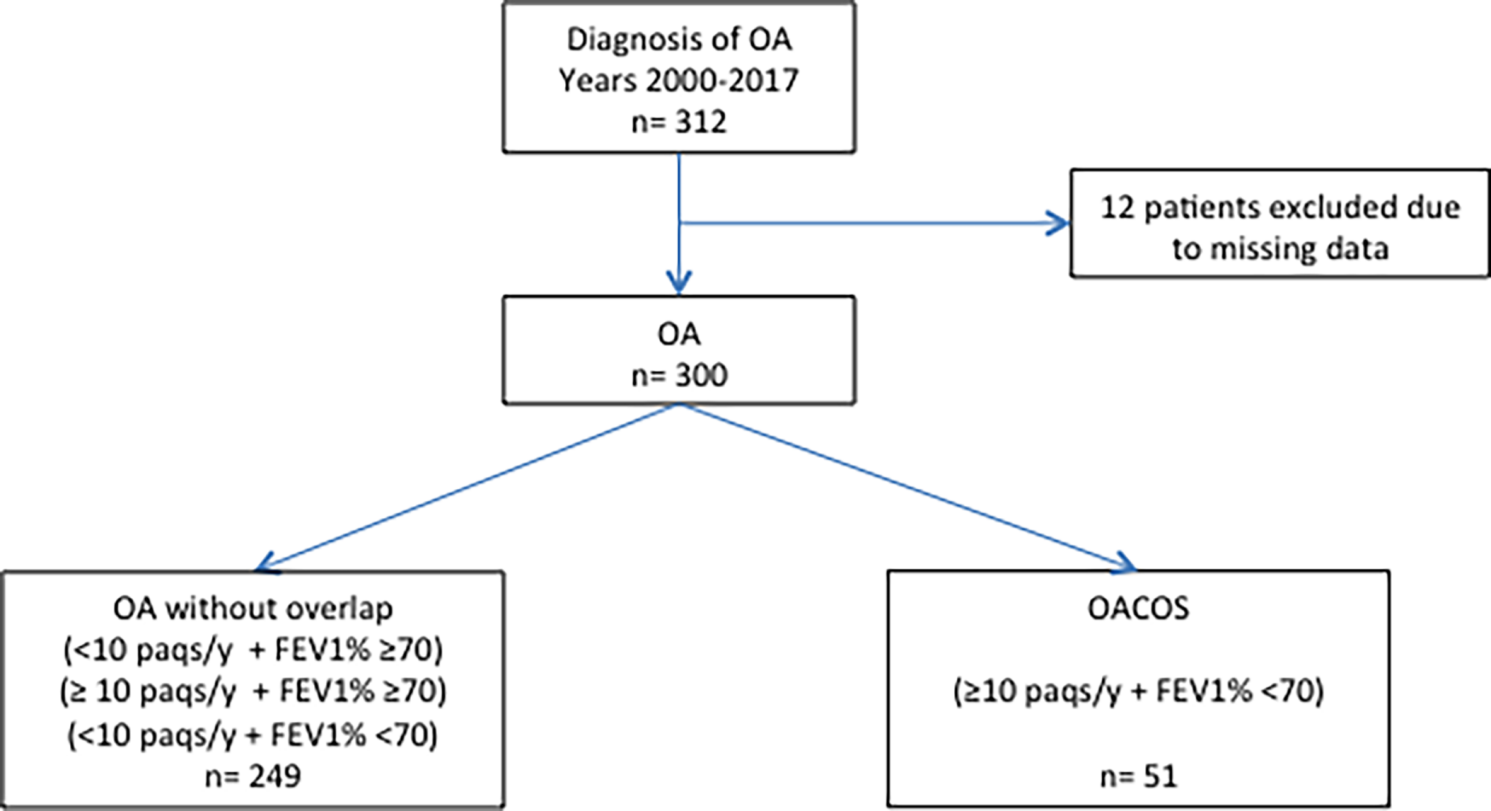
Study population. OA: occupational asthma; OACO: occupational asthma COPD overlap; FEV1: forced expiratory volume in the first second; FVC: forced vital capacity.

A total of 312 individuals were diagnosed with OA during the study period. Eight patients were excluded due to missing data in key variables such as smoking habits or FEV_1_/FVC. Therefore, 304 subjects were included, of which 262 (86.2%) patients were classified as OA and 42 (13.8%) as OACO (Fig 1).

Seventy one (27.1%) subjects with OA had a smoking history greater than 10 pack-years while 191 (72.9%) were non-smokers or had a smoking history lower than 10 pack-years.

#### Clinical characteristics

The clinical characteristics of the study subjects are summarized in Table 1.

**Table 1:** Clinical characteristics of the study population.

	OA	OACO	Missing values	p
n	262	42		
Age (y)	39.5 ± 10.7	52.0 ± 9.0	0	**<0.001**
Sex, n (%) Male	175 (66.8)	32 (76.2)	0	0.23
BMI (kg/m^2^)	28.2 ± 5.7	27.9 ± 5.5	54	0.78
Smoking Habits, n (%)			0	**<0.001**
Never smoker	116 (44.3)	0 (0)		
Ex-smoker	56 (21.4)	19 (45.3)		
Current smoker	90 (34.4)	23 (54.8)		
Cumulative smoking (PY)	6.5 ± 10.6	28.6 ± 15.8	0	**<0.001**
Duration of symptoms before visit (y)	3.4 ± 4.4	5.2 ± 7.1	0	0.1
Duration of expsoure before symptoms (y)	5.8 ± 7.5	12.7 ± 12.8	0	**0.001**
Duration of exposure before first visit (y)	9.4 ± 9.0	18.1 ± 13.8	0	**0.001**
Subjects taking ICS	146 (55.7)	40 (95.2)	0	**0.001**
Dose ICS (μg)	420.2 ± 454.6	839.3 ± 499.1	0	**<0.001**
Atopy, n (%)	218 (86.5)	23 (63.9)	16	**0.001**
Rhinitis, n (%)	61 (23.3)	5 (12.2)	1	0.07
FEV_1_ at baseline (% predicted)	92.4 ± 15.9	62.2 ± 15.1	0	**<0.001**
FEV_1_ baseline (L)	3.3 ± 0.9	1.9 ± 0.5	0	**<0.001**
FEV_1_/FVC at baseline	76.5 ± 8.9	56.8 ± 9.2	2*	**<0.001**
Type of occupational agent n(%)			33	**0.03**
LMW	120 (52.0)	28 (70.0)		
HMW	111 (48.1)	12 (30.0)		
Type of reaction to SIC, n (%)			30	**0.03**
Immediate reaction	99 (42.3)	9 (22.5)		
Non-immediate reaction	104 (44.4)	26 (65.0)		

Data are presented as mean ± standard deviation or, n (%). List of abbreviations: y, years; BMI, body max index; PY, Pack-year; ICS, inhaled corticosteroids; FEV_1_, forced expiratory volume in the first second; FVC, forced vital capacity; LMW, low-molecular-weight agents; HMW, high-molecular weight agents; SIC, specific inhalation challenge.

*, Two subjects were unable to perform reliable FVC maneuvers. These subjects had a FEV_1_ of 77% and 93% of predicted respectively and their smoking history was <10 pack years.

Subjects with OACO were older than subjects with OA (52.0 ± 9.0 vs 39.5 ± 10.7 years; p<0.001). By definition, OACO subjects were smokers who had a greater airflow obstruction than OA subjects. A higher percentage of OACO subjects were treated with ICS at higher doses than OA subjects (839.3 ± 499.1 vs 420.2 ± 454.6 μg/day respectively; p<0.001). Subjects in the OACO group had been exposed to the offending agent (18.1 ± 13.5 vs 9.4 ± 9.0; p<0.001) for a longer duration time than OA patients.

OACO subjects were predominantly exposed to low molecular agents (70.0% vs 52.0%; p = 0.03) compared to OA subjects and experienced more often non-immediate asthmatic reactions (42.3% vs 22.5%; p = 0.03) than OA subjects.

#### Functional and inflammatory characteristics of subjects with OACO and OA

OACO subjects presented lower baseline PC_20_ values than OA subjects (geometric mean [IC 95%], 0.7 [0.4–1.3] mg/ml vs 4.8 [3.8–6.1] mg/ml; p<0.001). Exposure to the offending agent did not induce a different change in airway hyperresponsiveness between OA and OACO groups (1.5 [0.7–3.5] vs 2.4 [1.9–3.1] (p = 0.23) respectively).

OA subjects presented higher blood eosinophil counts than OACO subjects (0.03 x10^6^ ± 0.03 cells/μL vs 0.02 ± 0.02 x10^6^ cells/μL; p = 0.001). No differences were found regarding blood neutrophil counts between OA and OACO subjects (0.64 x10^6^ ± 0.40 cells/μL vs 0.63 ± 0.08 x10^6^ cells/μL; p = 0.91).

**Fig 2:**
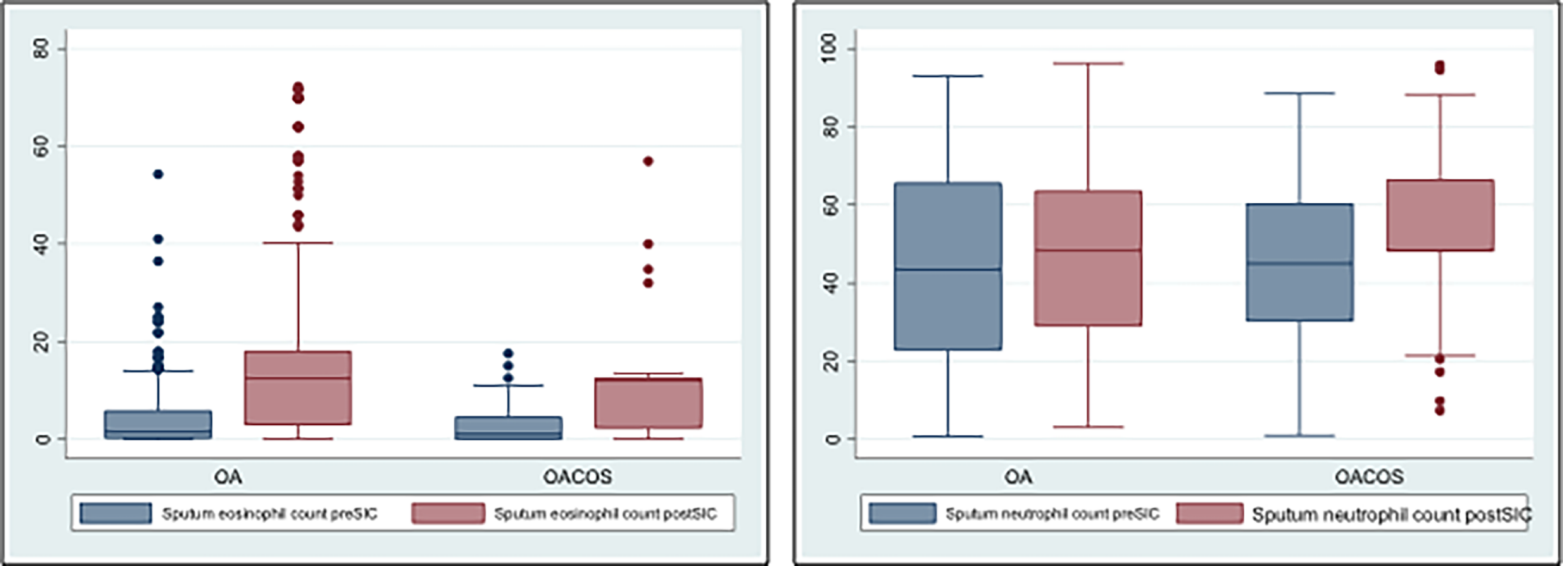
Sputum eosinophil and neutrophil counts before and after SIC in OA and OACO patients. OA: occupational asthma; OACO: occupational asthma COPD overlap; SIC: specific inhalation challenge.

Subjects in the OA group presented an increase in their sputum eosinophil counts after SIC (median [IQR], 4.0% [12.7]) while subjects in the OACO group did not (0.5% [6.0], p<0.001) (Fig 2).

There was no difference between steroid-naïve and steroid-treated subjects with OA (without ACO) before (1.3 (5.0)% and 1.0 (4.0) %) and after SIC (6.4(17.20)% and 6.5(17.5) % respectively). There was no difference in the level of sputum eosinophil counts after SIC in ACO subjects treated with low dose ICS (157.9± 223.0 μg) vs. moderate to high dose (976.3±288.6 μg): 2.3 (3.0)% vs. 2.4 (6.6)% respectively.

We did not observe any difference in sputum eosinophil counts between OACO smokers and OACO ex-smokers before (1.0[3.0] vs 1.85 [5.0]) and after (2.4 [2.3] vs 2.0 [9.4]) SIC.

There was no statistically significant difference in sputum neutrophil counts between OA and OACO groups before (median [IQR], 41.8% [41.2] vs 51.0% [46.0] p = 0.16) or after SIC (51.5% [29.7] vs 56.8% [43.8] p = 0.14) (Fig 2).

We did not observe any statistical difference between the inflammatory phenotype of the two groups. Although the neutrophilic phenotype was slightly higher among the ACOS group (21.4%) compared to the OA group (13.2%), it did not reach statistical significance (p = 0.6).

The inflammatory characteristics of OA and OACO subjects are presented in Table 2.

**Table 2:** Characterization on the airway inflammation on the study population.

	OA	Missing values	OACO	Missing values	p
n	262		42		
Blood Eosinophils (10^9^/L)	0.03 ± 0.03	88	0.02 ± 0.02	18	**<0.001**
Blood Neutrophils (10^9^/L)	0.64 ± 0.40	88	0.63 ± 0.08	18	0.91
PC_20_ (mg/mL), GM (IC 95%)					
Baseline	4.8 (3.8–6.1)	2	0.7 (0.4–1.3)	18	**<0.001**
Post-SIC	2.4 (1.9–3.1)	58	1.5 (0.7–3.5)	26	0.23
PC_20_ baseline/ PC_20_ post SIC	2.1 (1.8–2.6)	62	0.8 (0.5–1.2)	26	**<0.001**
Sputum TCC (10^6^ cells/g), median (IQR)					
Baseline	1.8 (2.9)	30	1.4 (2.5)	14	0.6
Post-SIC	3.2 (6.2)	39	2.1 (2.1)	19	**0.04**
Difference	1.1 (5.8)	39	0.1 (2.1)	19	**0.04**
Sputum eosinophils (%), median (IQR)					
Baseline	1.2 (4.0)	65	1.1 (4.3)	14	0.9
Post-SIC	6.5 (17.0)	59	2.3 (3.5)	19	**0.02**
Difference	4.0 (12.7)	77	0.5 (6.0)	19	**0.001**
Sputum neutrophils (%), median (IQR)					
Baseline	41.8 (41.2)	65	51.5 (29.7)	14	0.16
Post-SIC	51.0 (46.0)	59	56.8 (43.8)	19	0.14
Difference	3.3 (35.5)	77	10.2 (28.0)	19	0.77

Data are presented as mean ± standard deviation, geometric mean (GM) (IC 95%) or, median (interquartile range [IQR]). List of abbreviations: SIC, specific inhalation challenge; TCC, total cell count.

#### Adjusted analysis comparing OACO and OA groups

In the multivariate regression model, the only variables showing an independent association with OACO were older age (OR [95% CI]: 1.10 [1.05; 1.14]) and a higher ICS dose (OR [95% CI]: 5.20 **[**1.77; 16.48]). In contrast atopy was independently associated with OA diagnosis (OR [95% CI]: 0.30, [0.12; 0.65]) (Table 3).

**Table 3:** Regression model. Variables independently related to OACO diagnosis.

	OR	(95%CI)
Sex, Men	0.73	(0.23; 1.90)
Age at diagnosis	1.10	(1.05; 1.14)
ICS dose, ≥500 mcg	5.20	(1.77; 16.48)
Atopy	0.19	(0.07; 0.62)
Duration of exposition before symptoms (≥ 10 years)	1.73	(0.65; 3.99)

List of abbreviations: ICS, inhaled corticosteroids.

## Discussion

To our knowledge, this study is the first to identify and characterize subjects with ACO among patients with OA. OACO subjects were older, treated with higher doses of ICS and less atopic than subjects with OA besides having by definition a greater airflow obstruction and a greater smoking history than OA subjects.

In the present study, 13.8% of the subjects who received a diagnosis of OA had criteria for ACO. Different studies have established a prevalence of ACO that ranges from 12.1 to 55.2% among COPD patients, and 13.3 to 61.0% among subjects with asthma[3,6,8]. Although our results are consistent with the previous published prevalences, the proportion of subjects with ACO in OA seems to be at the lower end of previously published prevalences in the asthmatic population. This may be explained, at least in part, by the stringent criteria applied in our study.

Our results are consistent with a recent longitudinal study[13] performed in 188 asthmatic subjects. One hundred and fifty-four subjects were considered asthmatics (19 showed airflow limitation, FEV_1_/FVC <70% with <10 pack-years) and 34 were labeled as ACO (≥10 pack-years and FEV_1_/FVC <70%). Subjects with ACO or asthmatics with airflow limitation were mostly men, older, on higher doses of ICS, and less atopic than asthmatics without airflow obstruction.

Large epidemiological studies have compared ACO, defined as COPD individuals with a previous diagnosis of asthma, to COPD patients[4–6]. ACO subjects tended to be younger, predominantly female, with better lung function, lower smoking exposure, and a predominantly eosinophilic inflammation, compared to COPD patients[24]. As expected, the ACO population characterized among a COPD population differs from an ACO population defined within an asthmatic population.

Several studies have assessed the changes in sputum cell counts before and after SIC in subjects with OA[25,26]. While the majority of subjects with OA show an increase in sputum eosinophil counts; an increase in neutrophil count has also been reported[25]. Previous studies found higher blood neutrophil counts and higher IL-6 values[13] in ACO subjects compared to asthmatic subjects. However, those studies did not show differences in blood eosinophil counts between ACO and asthmatics[12,13]. Subjects with OACO tended to have slightly higher neutrophil counts than subjects with OA, although this difference did not reach statistical significance. The small sample size of the OACO group along with the high number of missing values did not allow us to confirm the hypothesis that OACO group showed a greater neutrophilic inflammation than the OA group. Although we did not find any difference in baseline values of sputum eosinophil counts between OA and OACO subjects, OA subjects presented a greater increase in sputum eosinophil counts after SIC than OACO subjects. Furthermore, blood eosinophils were slightly higher in subjects with OA than in OACO subjects. The OACO group of subjects did not show any increase in their sputum eosinophil count after exposure to the offending agent even if they experienced a 20% fall in FEV_1_.

OACO subjects were treated with higher doses of ICS than OA subjects. Although we could not compare the OACO subjects with and without inhaled corticosteroids (ICS) since only two of them did not take ICS, we did not observe any difference between steroid-naïve and steroid-treated subjects with OA in sputum eosinophil counts before and after SIC. Furthermore, we did not find any difference in the level of sputum eosinophil counts after SIC in ACO subjects treated with low dose ICS vs. moderate to high dose. Therefore, we do not believe that treatment with ICS alone explains the minimal increase of eosinophilic airway inflammation after SIC in ACO subjects.

Smoking has been shown to suppress airway inflammation induced after allergen challenge in both mice[27] and humans[28]. Therefore, it is likely that lack of increase in sputum eosinophil count observed in OACO subjects could be related to the smoking exposure of this group. We had previously showed that non-eosinophilic responders with OA seemed to have a poorer prognosis than eosinophilic responders[29]. Whether the prognosis of OACO patients is poorer than OA subjects remains to be shown.

The results of the present study are consistent with studies that described ACO patients among asthmatic individuals. It has been widely emphasized that ACO subjects identified from COPD populations could benefit from adding ICS, based on the efficacy of this treatment to decrease eosinophilic airway inflammation[8]. In contrast, OACO identified among OA subjects have shown to have a mixed eosinophilic-neutrophilic airway inflammation, but, nevertheless, were already receiving higher ICS doses, probably related to a greater airflow obstruction.

The strength of this study is to gather a unique population of subjects with OA objectively diagnosed with SIC with functional and inflammatory characteristics before and after exposure to the offending agent. However, our study suffers from some limitations, basically the retrospective nature of the study and the absence of data regarding symptoms and exacerbations prevented us to perform a follow-up of those subjects to assess their long-term prognosis.

## Conclusions

Approximately 14% of subjects with OA present characteristics of OACO. OACO constitutes a distinct clinical and inflammatory phenotype. Whether this group of subjects should be managed in a different way and present a different prognosis remains to be determined.

## Supporting information

S1 File Datase. (XLSX)
