# Correction: Clinical and inflammatory characteristics of Asthma-COPD overlap in workers with occupational asthma

**DOI:** 10.1371/journal.pone.0195648

**Published:** 2018-04-04

**Authors:** Iñigo Ojanguren, Gregory Moullec, Jad Hobeika, Marc Miravitlles, Catherine Lemiere

Fig 1 and Fig 2 are based on an earlier version of the results. Please view the corrected Fig 1 and Fig 2 here.

**Fig 1:**
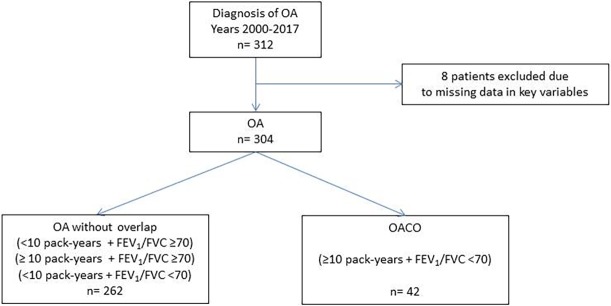
Study population. OA: occupational asthma; OACO: occupational asthma COPD overlap; FEV1: forced expiratory volume in the first second; FVC: forced vital capacity.

**Fig 2:**
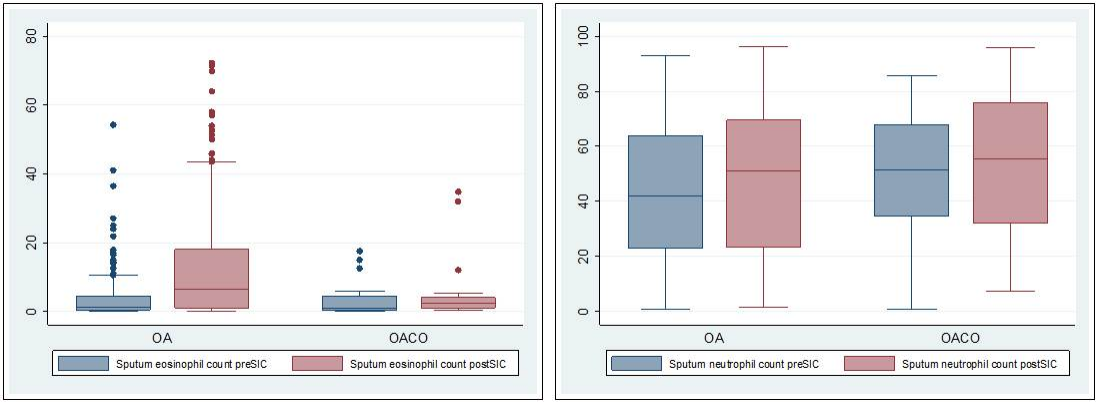
Sputum eosinophil and neutrophil counts before and after SIC in OA and OACO patients. OA: occupational asthma; OACO: occupational asthma COPD overlap; SIC: specific inhalation challenge.

## References

[1] Ojanguren I, Moullec G, Hobeika J, Miravitlles M, Lemiere C (2018) Clinical and inflammatory characteristics of Asthma-COPD overlap in workers with occupational asthma. PLoS ONE 13(3): e0193144 https://doi.org/10.1371/journal.pone.0193144 29499062 10.1371/journal.pone.0193144 PMC5834173

